# Screening Suitable Reference Genes for Normalization in Reverse Transcription Quantitative Real-Time PCR Analysis in Melon

**DOI:** 10.1371/journal.pone.0087197

**Published:** 2014-01-27

**Authors:** Qiusheng Kong, Jingxian Yuan, Penghui Niu, Junjun Xie, Wei Jiang, Yuan Huang, Zhilong Bie

**Affiliations:** Key Laboratory of Horticultural Plant Biology, Ministry of Education/College of Horticulture and Forestry, Huazhong Agricultural University, Wuhan, P. R. China; Wuhan University, China

## Abstract

Melon (*Cucumis melo*. L) is not only an economically important cucurbitaceous crop but also an attractive model for studying many biological characteristics. Screening appropriate reference genes is essential to reverse transcription quantitative real-time PCR (RT-qPCR), which is key to many studies involving gene expression analysis. In this study, 14 candidate reference genes were selected, and the variations in their expression in roots and leaves of plants subjected to biotic stress, abiotic stress, and plant growth regulator treatment were assessed by RT-qPCR. The stability of the expression of the selected genes was determined and ranked using geNorm and NormFinder. geNorm identified the two most stable genes for each set of conditions: *CmADP* and *CmUBIep* across all samples, *CmUBIep* and *CmRPL* in roots, *CmRAN* and *CmACT* in leaves, *CmADP* and *CmRPL* under abiotic stress conditions, *CmTUA* and *CmACT* under biotic stress conditions, and *CmRAN* and *CmACT* under plant growth regulator treatments. NormFinder determined *CmRPL* to be the best reference gene in roots and under biotic stress conditions and *CmADP* under the other experimental conditions. *CmUBC2* and *CmPP2A* were not found to be suitable under many experimental conditions. The catalase family genes *CmCAT1*, *CmCAT2*, and *CmCAT3* were identified in melon genome and used as target genes to validate the reliability of identified reference genes. The catalase family genes showed the most upregulation 3 days after inoculation with Fusarium wilt in roots, after which they were downregulated. Their levels of expression were significantly overestimated when the unsuitable reference gene was used for normalization. These results not only provide guidelines for the selection of reference genes for gene expression analyses in melons but may also provide valuable information for studying the functions of catalase family genes in stress responses.

## Introduction

Melon (*Cucumis melo* L.), a member of the Cucurbitaceae family, ranks as the 9th most cultivated horticultural crop in terms of total world production [1]. In addition to its significant economic value, melon is also an attractive model for the study of valuable biological characteristics, such as fruit ripening, sex determination, and phloem physiology. The full sequence of melon genome is readily available, which greatly facilitates the identification of genes underlying certain traits and the elucidation of mechanisms that regulate relevant characteristics [2].

Gene expression analysis is a major experimental approach in functional genomics studies. It can be used to evaluate complex regulatory gene networks [3]. Currently, reverse transcription quantitative real-time PCR (RT-qPCR) is a standard tool for the quantification of levels of gene expression. It is rapid, sensitive, and specific [4]. However, the accuracy of the results obtained by this method depends on exact transcript normalization using stably expressed gene, the reference gene, which is presumed to be representative of the cDNA concentration in each sample and subject to the same errors during cDNA preparation as target gene [5], [6]. The reliability and accuracy of RT-qPCR is lost if inappropriate reference gene is selected [7]. The selection of suitable reference gene is vital to RT-qPCR analysis.

According to the generally accepted criteria, the ideal reference gene is stably expressed (or at least varies in expression only slightly) across the investigated sample sets and has a level of expression comparable to that of target gene or genes [8]. Lists of suitable reference genes have been identified for many crops, especially for model plants [9]. However, no universal reference gene exists. This is because the expression of putative reference genes varies across different sets of organs and different experimental conditions [5], [10]. It is likely that one or more genes are constitutively expressed across a specific organ in a specific environment [8]. In this way, the selection and systematic validation of reference genes must be conducted before any meaningful RT-qPCR analyses can be performed [11].

The validation of reference genes has received very little attention in melons. Arbitrarily chosen classic housekeeping genes have been used as references for normalization in RT-qPCR analyses involving melons. *Actin* is the most widely used reference gene in melons [12]–[14]. It has been used in melons subjected to Fusarium wilt infection [15], [16], salt stress [17], and different forms of nitrogen [18]. *Cyclophilin* has also served as reference gene in melon fruits and other organs [19], [20]. In addition, *ubiquitin* was identified to be the suitable reference gene for normalizing the expressions of phosphorus responsive genes in roots [1] and *ribosomal protein L2* was demonstrated to have high transcript stability in stems infected with Fusarium wilt [21]. The reference genes currently in use are the only two that have been validated in melons. The use of non-validated reference genes can dramatically affect normalization and may introduce errors [5]. In addition, classical reference genes including *actin* have been proven to be unsuitable because their levels of transcriptions are regulated differently in different experimental settings and tissues [22]. Considering the increasing interest in functional genomics of melons that has arisen in response by the recent availability of genome and transcriptome data, the requirements for appropriate reference genes for expression normalization have become much more stringent.

Catalase (*CAT*) genes, which encode a small family of proteins and catalyze the decomposition of hydrogen peroxide (H_2_O_2_) to water and oxygen, play important roles in controlling homeostasis of reactive oxygen species (ROS). ROS have been reported to be as toxic and to act as signal molecules in plant responses to environmental stresses [23], [24]. Melons are susceptible to many biotic and abiotic stresses. These negatively affect growth and yield. Understanding the patterns of expression of genes associated with stress responses provide insight into the gene regulation mechanisms and allow scientists improve the stress tolerance of melons. In the present study, 14 reference genes reported in previous studies were selected as candidates, the levels of expression of their melon homologues were evaluated by RT-qPCR in the leaves and roots under various stresses and growth regulator treatments, and the stability of expression was calculated and compared using the algorithms of geNorm and NormFinder [8], [25]. The expression profiles *CAT* family genes were examined in roots infected with Fusarium wilt by the identified reference genes. These results may provide guidelines for the use of reference genes suitable for the quantification of gene expression in melons using RT-qPCR.

## Materials and Methods

### Plant materials and treatments

‘*Elizabeth*’ (var. *inodorus*), a commercial F_1_ hybrid, was used in the present study. The seeds were first sterilized with 1.5% sodium hypochlorite, soaked in distilled water for 4 h, and then stored at 30°C for germination. The germinated seedlings were grown in pots containing sterilized vermiculite and cultured under 14 h artificial-light at 25°C and 10 h-dark at 18°C with 65–75% relative humidity in a controlled environment chamber. Hoagland nutrient solution was added at 3-day intervals. Seedlings at the second true leaf stage were used for the following treatments.

The devastating diseases of Fusarium wilt and bacterial fruit blotch frequently occur in melons. They were used here to induce biotic stress. Artificial inoculation of Fusarium wilt was carried out with a 3×10^6^ spore/mL suspension of *Fusarium oxysporum* f. sp. *melonis* (*F.o.m.*) races 1 as described by Oumouloud et al. [26]. Artificial inoculation of bacterial fruit blotch was conducted as described by Bahar et al. [27]. Briefly, melon seedlings were sprayed with a 10^8^ cfu/mL suspension of *pslbtw20* strain (*Acidovorax avenae* subsp. *Citrulli*) until runoff. Roots and leaves were sampled at 3 days post inoculation and the remaining seedlings were kept till the typical symptoms of Fusarium wilt, or bacterial fruit blotch were visible to confirm the success of artificial inoculation.

Low temperature, salinity, and drought often cause great losses in melon production. These were used here to induce abiotic stress. For low-temperature treatment, the seedlings were kept at 4±1°C for 3 h. For salt and drought stress treatments, seedlings were cultivated hydroponically in Hoagland nutrient solution supplemented with 300 mM NaCl for 5 h or 15% PEG 6000 for 24 h. Roots and leaves were sampled for each of the abiotic stress treatments.

For plant regulator treatments, seedlings were sprayed with solutions of abscisic acid (ABA), salicylic acid (SA), methyl jasmonic acid (MeJA), gibberellin (GA), 1-naphthlcetic acid (NAA), or ethrel at a concentration of 100 µM, and their leaves were sampled 3 h later.

Roots and leaves were taken from seedlings that had not undergone any treatments at second true leaf stage. These served as controls. Each experiment was repeated in triplicate and each replicate contained 15 seedlings. The roots and leaves of five randomly selected plants were collected for each treatment, immediately frozen in liquid nitrogen and kept at −80°C for subsequent RNA extraction.

A total of 18 samples were used to investigate the stability of expression of the candidate genes. To find more stable genes in the organs in question or under the experimental conditions in question, the total sample set was subdivided into different subsets, biotic stress subset (6 samples including roots and leaves from controls and seedlings infected with Fusarium wilt or bacterial fruit blotch), abiotic stress subset (8 samples including roots and leaves from controls and seedlings treated with low temperature, salinity or drought), plant growth regulator treatment subset (7 samples including leaves from controls and seedlings sprayed with ABA, SA, MeJA, NAA, GA or ethrel), root subset (6 samples including roots from controls and seedlings treated with biotic or abiotic stresses), and leaf subset (12 samples including leaves from controls and seedlings treated with biotic stresses, abiotic stresses or plant growth regulators).

### RNA isolation and cDNA synthesis

The eleven golden rules of RT-qPCR were used as guidelines for RNA isolation, cDNA synthesis, and subsequent RT-qPCR analyses [28]. Total RNA was extracted using the TRIzol Reagent (Invitrogen) according to the manufacturer's instructions. RNA integrity was checked by electrophoresis in 2% (w/v) agarose gel and then quantity and purity were determined with a NanoDrop 2000 spectrophotometer (Thermo Scientific). Only high-quality samples with A_260_/A_280_>1.8 and A_260_/A_230_>2.0 were used for cDNA synthesis. Genomic DNA elimination and cDNA synthesis were conducted using a PrimeScript RT Reagent Kit with gDNA Eraser (Perfect Real Time) (TaKaRa) for manual description. For each sample, 1 µg total RNA was used for each 20 µL reverse transcription reaction system.

### Selection of candidate reference genes and primer design

In order to identify suitable reference genes for melons, 14 candidates that had been reported in other crops or frequently used in melons were selected. These included β-actin (*ACT*), elongation factor 1-α (*EF1α*), ubiquitin extension protein (*UBIep*), protein phosphatase 2A regulatory subunit A (*PP2A*), 60S ribosomal protein L36a/L44 (*RPL36aA*), α-tubulin (*TUA*), actin 7a (*ACT7*), glyceraldehyde-3-phosphate dehydrogenase C2 (*GAPC2*), GTP-binding nuclear protein (*RAN*), ribosomal protein L (*RPL*), ribosomal protein L2 (*RPL2*), ADP-ribosylation factor 1 (*ADP*), ubiquitin-conjugating enzyme E2 (*UBC2*), and 18S ribosomal RNA (*18SrRNA*).

To obtain melon orthologous sequences of the candidate genes, blastn searches were performed using *Arabidopsis* genes as queries on melon unigenes in the Melonomics database (https://melonomics.net/). Melon homologue sequences with the best matches were retrieved and submitted to Primer3 (http://bioinfo.ut.ee/primer3-0.4.0/) for primer design. Product size was set at the range of 80–150 bp and default values for other parameters were adopted. Candidate primer pair sequences were then used as queries to blast against melon unigenes in the Melonomics database to confirm their specificity *in silico*. The specificity of the PCR amplification product for each primer pair was further determined by electrophoresis in 2% (w/v) agarose gel and melting-curve analysis. Finally, the abbreviation of the species name *Cucumis melo*, “*Cm*,” was added as a prefix to the name of each selected gene to specify that this was an orthologous gene in melons. Information regarding the selected genes and their primer pairs is listed in Table 1.

**Table 1:** Descriptions of melon candidate reference genes for RT-qPCR analysis.

Gene name	Gene description	Unigene ID^1^	Arabidopsis homolog 2	E-value	Primer sequence^3^	Product size (bp)	E (%)^4^
*Cm18SrRNA*	18S ribosomal RNA	MU60789	AT3G41768	0	F:CGAGTCTGGTAATTGGAATGAGTA	137	95.8
					R:CTACGAGCTTTTTAACTGCAACAA		
*CmACT*	β-actin	MU51303	AT3G18780	0	F:CCTGGTATCGCTGACCGTAT	133	96.7
					R:TACTGAGCGATGCAAGGATG		
*CmACT7*	Actin 7a	MU43164	AT5G09810	0	F:TCGTTCCTTCCTTCCTTCATTC	120	104.8
					R:AGCCTTCACCATTCCAGTTC		
*CmADP*	ADP ribosylation factor 1	MU47713	AT1G23490	1e-125	F:ATATTGCCAACAAGGCGTAGA	93	99.3
					R:TGCCCGTAAACAAGGGATAAA		
*CmEF1α*	Elongation factor 1-α	MU45992	AT5G60390	0	F:CCACGAGTCTCTCCCAGAAG	83	97.2
					R:CACGCTTGAGATCCTTGACA		
*CmGAPC2*	Glyceraldehyde-3-phosphate dehydrogenase C2	MU54550	AT1G13440	0	F:CTGCCAAGGCTGTAGGTAAA	100	103.6
					R:GTAAGGTCAACAACGGAGACA		
*CmPP2A*	Protein phosphatase 2A regulatory subunit A	MU43409	AT1G69960	1e-104	F:GGGACCGATGTGTGATCTCT	129	95.6
					R:GTTAGCCCATTCGTGTGGTT		
*CmRAN*	GTP-binding nuclear protein	MU45556	AT5G20020	1e-106	F:TGCACCCTTTGGACTTCTTC	104	98.7
					R:GATGTAGTAGCCATCCCGTAAAC		
*CmRPL*	Ribosomal protein L	MU45916	AT1G04480	3e-97	F:CGACAATACTGGAGCCAAGAA	100	99.4
					R: CATCACCATATCTCCCACACAA		
*CmRPL2*	Ribosomal protein L2	MU53752	ATCG00830	0	F:GAGCCGTAGACAGTCAAGTAAA	111	96.0
					R:CTCTATGCCCTGCGGTAATG		
*CmRPL36aA*	60S ribosomal protein L36a/L44	MU54545	ATCG00760	5e-31	F:GAAGGGCAATGACACGAAAT	127	97.6
					R: CAATATTCGGGGGATCCTTT		
*CmTUA*	α-Tubulin	MU45968	AT1G04820	0	F: GCGGTGCTTCTAGACAATGA	100	97.3
					R:CCTGAGATACAAGACGGTTGAG		
*CmUBC2*	Ubiquitin conjugating enzyme E2	MU64017	AT2G02760	3e-79	F:TTAGTGGTGCTCCCCAAGAC	142	104.1
					R: CGGACTGTTGGTGGCTTATT		
*CmUBIep*	Ubiquitin extension protein	MU54467	AT2G36170	4e-56	F:AAGTGTGGACACAGCAACCA	132	103.8
					R:AAGCCAAATGGCTCTAAGCA		

“1” : Melon unigene ID in Melonomic database (https://melonomics.net/genome/) or Cucurbit Genomics Database (http://www.icugi.org/cgi-bin/ICuGI/index.cgi);

“2” : Arabidopsis gene ID in TAIR database (http://www.arabidopsis.org/);

“3” : F, forward primer; R, reverse primer;

“4” : E, amplification efficiency.

### RT-qPCR analysis

RT-qPCR was carried out on a LightCycler480 System (Roche) using SYBR Premix Ex Taq (TaKaRa). Reactions were performed in a total volume of 10 µL containing 100 ng of cDNA template, 0.2 µM of each amplification primer, and 1×SYBR Premix Ex Taq. The PCR cycling conditions were as follows: 95°C for 30 s, followed by 40 cycles of 95°C for 5 s, 55°C for 30 s, and 72°C for 30 s. Melting curves were recorded after 40 cycles to confirm primer specificity by heating from 65°C to 95°C. Negative controls were included for each pair of primers, and each RT-qPCR reaction was performed in two technical and three biological replicates. The efficiency of PCR amplification was derived from a standard curve generated by five 10-fold serial dilution points of cDNA mixed by all samples.

### Data analysis

The expression levels of the tested reference genes were determined by Cp (crossing point) values. To describe the expression levels and variations of the candidate reference genes, a boxplot was draw using R package (http://www.R-project.org/). The amplification efficiency of each RT-qPCR primer pair was calculated from the standard amplification cure using the equation of E (%) = (10^−1/slope^−1)*100, where slope is the standard curve slope calculated by the LightCycle 480 system. The publicly available tools geNorm and NormFinder were used to assess the expression stability of selected genes [8], [25]. geNorm calculates the gene expression stability measure (M) for each gene based on the average pairwise expression ratio between a particular gene and all other control genes. The gene with the most stable expression is given the lowest M value. By stepwise exclusion of the least stable gene, geNorm determines the two most stable reference genes for normalization or a combination of multiple stable genes and uses these results to calculate a robust normalization factor [25]. NormFinder, taking into account intra- and inter-group variations, evaluates the expression stability of each single reference gene. Genes that are more stably expressed are given lower expression stability values [8]. For the both tools, the input data is supposed to be on a linear scale. Before calculation, the Cp values were converted into relative quantities according to the formula Q = 2^ΔCp^. Here, ΔCp is the lowest Cp value minus the Cp value of the sample tested.

### Normalization of catalase family genes

In order to validate the reliability of the identified reference genes, relative expression patterns of melon catalase family genes were analyzed in response to Fusarium wilt infection in roots. To identify the catalase family genes in the melon genome, the Hidden Markov Model of catalase family (PF00199) was downloaded from the Pfam database (http://pfam.sanger.ac.uk/) and used as a query to search against the melon proteins CM_3.5 (https://melonomics.net/) using HMMER [29]. The primers were designed using Primique, which can be used to design the specific PCR primers for each gene in the family [30]. Information regarding the melon catalase family genes and their primers is summarized in Table S1. Roots at 0 d, 1 d, 3 d, and 5 d post inoculation with Fusarium wilt were sampled as described in the materials and treatments section of this paper. The levels of expression of melon catalase family genes were measured using RT-qPCR and calculated using 2^−ΔΔCp^. The most and least stable genes identified by geNorm and NormFinder served as reference genes.

## Results

### Candidate reference genes selection and amplification specificity

A total of 14 genes that have been used in the studies of cucurbit and other crops were selected for use as candidate reference genes for melons. Based on the melon genome and unigene sequences, homologous gene sequences were acquired for melons by using sequence similarity searches. Primer3 software was used for primer design. It usually generates many candidate primer pairs per gene. The candidate primer pair sequences were then used as queries to blast against melon unigenes, and only the primer pairs that specifically matched the target unigene were selected. To further confirm the specificity of the primer pairs, the PCR amplification products were analyzed by agarose gel electrophoresis using genomic DNA and mixed cDNA as templates. The results are presented in Fig. S1. All primer pairs generated a single band with the desired size on cDNA templates. The primers of *CmACT7*, *CmUBC2*, and *CmUBIep* amplified larger products on genomic DNA templates, indicating the existence of introns in the amplified regions. Two bands were generated for *CmUBC2* on genomic DNA templates. This was probably due to the existence of pseudogenes in the genome. The results obtained by agarose gel electrophoresis not only confirmed the specificity of the primer pairs but also proved that there was no genomic DNA contamination in the cDNA samples. A melting curve analysis was also conducted to confirm the amplification specificity, and all the primer pairs listed in Table 1 exhibited a single peak with no visible primer-dimer formation (Fig. S2).

A standard curve was generated by five serial 10-fold dilutions of the mixed cDNA in a RT-qPCR assay to determine the amplification efficiency for each primer pair. The efficiency values of selected primers ranged from 95.6% for *CmPP2A* to 104.8% for *CmACT7* (Table 1), implying the RT-qPCR system in the study was efficient and specific to melon candidate gene amplification.

### Expression profiles of the candidate reference genes

**Figure 1:**
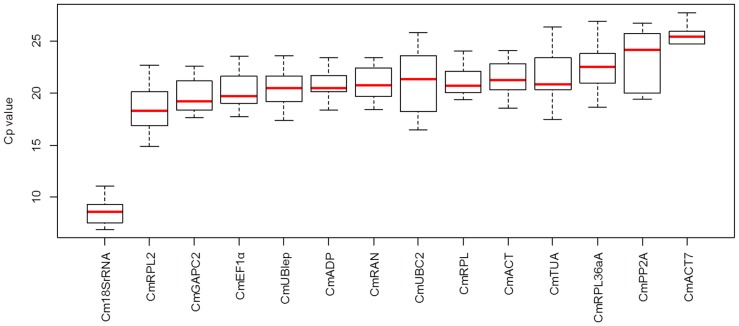
Expression profiles of melon candidate reference genes. Expression data are displayed as Cp values for each reference gene in all samples. The line across the box indicates the median. The box indicates the 25th and 75th percentiles. Whiskers represent the maximum and minimum values.

RT-qPCR was carried out to determine the expression profiles of the candidate reference genes. All samples for each candidate reference gene were run on the same plate to exclude technical variations. The expression levels of candidate reference genes are presented as the raw Cp values. The selected genes showed a relatively wide range of expression abundance and variation, as shown in box-and-whiskers plots (Fig. 1). *CmACT7* showed the lowest level of expression with the mean Cp value as high as 25.1 cycles. In comparison, *Cm18SrRNA* had the highest level of expression and an average Cp value of 8.6. For most of the candidate reference genes, average Cp values were within the range of 20 to 22 cycles. No candidate reference genes had constant expression levels among the tested samples. *Cm18SrRNA* and *CmRAN* showed the least variation in expressions among the tested samples, and *CmUBC2* had the most variation. This shows that it is important to screen appropriate reference genes via statistical methods in gene expression analyses involving melons.

### Expression stability of the candidate reference genes

**Figure 2:**
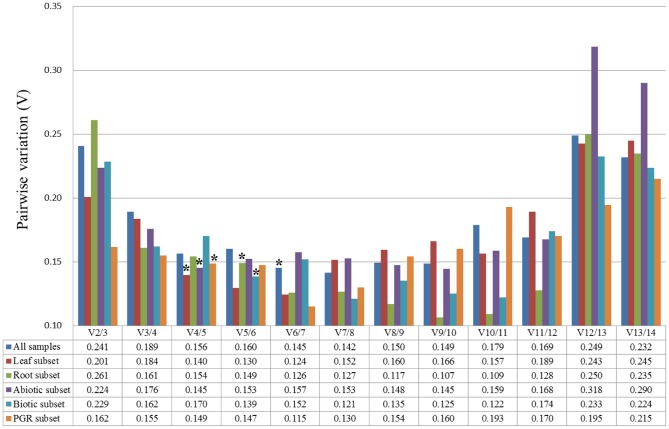
Pairwise variation analyses of candidate reference genes in different sample sets. Pairwise variation (V) was calculated using geNorm to determine the minimum number of reference genes required for accurate normalization in different sample sets. The asterisks indicate the value of pairwise variation less than the recommended 0.15 for each sample set. PGR subset represents the plant growth regulator treatment subset.

The most popular tools of geNorm and NormFinder were used to aid the selection of suitable reference genes from among the candidates. Those two programs evaluate gene expression stability using different algorithms. However, both programs consider genes with low expression stability values to be more stably expressed than those with high values. The expression stability of the candidate reference genes determined by geNorm and NormFinder are summarized in Tables 2 and 3, respectively. When all 18 samples were considered, *CmADP* and *CmUBIep* (M = 0.75) were ranked as the most stably expressed reference genes by geNorm, and *CmADP* was also ranked as the most stable reference gene by NormFinder. *CmPP2A* was identified as the least stably expressed gene by both geNorm and NormFinder. Usually, using multiple reference genes is more accurate and reliable for data normalization than using a single reference gene. The geNorm algorithm also calculates pairwise variation values (V) to determine the minimum number of reference genes required for accurate and reliable normalization. It is recommended that no additional genes be used for the normalization when *V_n/n+1_* is below 0.15. Pairwise variation analysis showed that at least six reference genes, including *CmADP*, *CmUBIep*, *CmRPL*, *CmRAN*, *CmACT* and *CmEF1α*, were needed for more reliable normalization across all the samples. This was because *V_6/7_* (0.145) was below the recommended cut-off value (Fig. 2).

**Table 2:** Expression stability of candidate reference genes ranked by geNorm in different sample sets.

All samples		Root subset		Leaf subset		Abiotic stress subset		Biotic stress subset		PGR subset^1^	
Gene	M^2^	Gene	M	Gene	M	Gene	M	Gene	M	Gene	M
*CmADP/CmUBIep* *	0.75	*CmUBIep/CmRPL* *	0.42	*CmRAN/CmACT* *	0.56	*CmADP/CmRPL* *	0.58	*CmTUA/CmACT* *	0.26	*CmRAN/CmACT* *	0.34
*CmRPL* *	0.79	*CmADP* *	0.68	*CmGAPC2* *	0.63	*CmRAN* *	0.68	*CmRPL* *	0.55	*CmEF1α* *	0.46
*CmRAN* *	0.84	*CmRAN* *	0.72	*CmADP* *	0.72	*CmUBIep* *	0.74	*CmUBIep* *	0.63	*CmGAPC2* *	0.56
*CmACT* *	0.87	*CmACT* *	0.78	*CmRPL*	0.76	*CmACT*	0.78	*CmRAN* *	0.74	*CmRPL*	0.66
*CmEF1α* *	0.94	*CmGAPC2*	0.86	*CmUBIep*	0.81	*CmEF1α*	0.86	*CmADP*	0.80	*CmADP*	0.76
*CmGAPC2*	1.01	*CmEF1α*	0.91	*CmEF1α*	0.86	*CmTUA*	0.97	*CmEF1α*	0.90	*CmUBIep*	0.81
*CmTUA*	1.08	*CmTUA*	0.97	*CmTUA*	0.98	*CmGAPC2*	1.06	*CmGAPC2*	0.96	*Cm18SrRNA*	0.90
*Cm18SrRNA*	1.18	*CmRPL36aA*	1.02	*Cm18SrRNA*	1.11	*CmACT7*	1.15	*CmACT7*	1.05	*CmTUA*	1.03
*CmACT7*	1.28	*Cm18SrRNA*	1.06	*CmACT7*	1.25	*Cm18SrRNA*	1.24	*Cm18SrRNA*	1.12	*CmACT7*	1.17
*CmRPL2*	1.44	*CmACT7*	1.12	*CmRPL2*	1.38	*CmRPL36aA*	1.37	*CmUBC2*	1.19	*CmRPL36aA*	1.37
*CmRPL36aA*	1.57	*CmRPL2*	1.21	*CmRPL36aA*	1.56	*CmRPL2*	1.51	*CmRPL2*	1.36	*CmRPL2*	1.52
*CmUBC2*	1.86	*CmPP2A*	1.53	*CmUBC2*	1.83	*CmPP2A*	1.93	*CmRPL36aA*	1.64	*CmUBC2*	1.71
*CmPP2A*	2.09	*CmUBC2*	1.81	*CmPP2A*	2.09	*CmUBC2*	2.26	*CmPP2A*	1.88	*CmPP2A*	1.93

“1” : PGR subset, plant growth regulator treatment subset;

“2” : M, stability measure;

“*” : represents the optimal reference genes determined by pairwise variation analyses which are presented in Fig. 2. The recommended threshold of 0.15 is adopted.

**Table 3:** Expression stability of candidate reference genes ranked by NormFinder in different sample sets.

All samples		Root subset		Leaf subset		Abiotic stress subset		Biotic stress subset		PGR subset^1^	
Gene	SV^2^	Gene	SV	Gene	SV	Gene	SV	Gene	SV	Gene	SV
*CmADP*	0.45	*CmRPL*	0.22	*CmADP*	0.45	*CmADP*	0.28	*CmRPL*	0.16	*CmADP*	0.42
*CmACT*	0.50	*CmACT*	0.35	*CmGAPC2*	0.50	*CmRAN*	0.56	*CmADP*	0.19	*Cm18SrRNA*	0.43
*CmRAN*	0.52	*CmUBIep*	0.37	*Cm18SrRNA*	0.51	*CmRPL*	0.57	*CmUBIep*	0.32	*CmRAN*	0.46
*CmRPL*	0.55	*CmGAPC2*	0.40	*CmRAN*	0.53	*Cm18SrRNA*	0.59	*CmRAN*	0.52	*CmGAPC2*	0.50
*Cm18SrRNA*	0.57	*CmADP*	0.46	*CmACT*	0.56	*CmACT7*	0.61	*CmTUA*	0.54	*CmACT*	0.53
*CmUBIep*	0.67	*CmEF1α*	0.48	*CmRPL*	0.56	*CmACT*	0.65	*Cm18SrRNA*	0.55	*CmRPL*	0.54
*CmEF1α*	0.72	*CmRAN*	0.54	*CmEF1α*	0.80	*CmEF1α*	0.73	*CmACT*	0.61	*CmUBIep*	0.54
*CmGAPC2*	0.75	*Cm18SrRNA*	0.66	*CmUBIep*	0.80	*CmGAPC2*	0.82	*CmUBC2*	0.84	*CmEF1α*	0.64
*CmACT7*	1.01	*CmACT7*	0.81	*CmRPL2*	1.10	*CmUBIep*	0.88	*CmEF1α*	0.90	*CmTUA*	1.10
*CmTUA*	1.01	*CmTUA*	0.83	*CmTUA*	1.11	*CmRPL36aA*	1.04	*CmGAPC2*	0.98	*CmACT7*	1.22
*CmRPL36aA*	1.31	*CmRPL36aA*	0.91	*CmACT7*	1.13	*CmTUA*	1.25	*CmACT7*	1.06	*CmRPL36aA*	1.24
*CmRPL2*	1.36	*CmRPL2*	1.28	*CmRPL36aA*	1.35	*CmRPL2*	1.48	*CmRPL2*	1.27	*CmRPL2*	1.35
*CmUBC2*	2.12	*CmPP2A*	2.08	*CmUBC2*	2.09	*CmPP2A*	2.65	*CmRPL36aA*	2.02	*CmUBC2*	1.67
*CmPP2A*	2.23	*CmUBC2*	2.27	*CmPP2A*	2.36	*CmUBC2*	2.79	*CmPP2A*	2.16	*CmPP2A*	2.07

“1” : PGR subset, plant growth regulator treatment subset;

“2” : SV, stability value.

To identify the more reliable reference genes under different experimental conditions, the samples were divided into 5 subsets according to the organ and treatment groups to screen for organ-specific and experimental-condition-specific reference genes.

In the root subset (6 samples), *CmRPL* and *CmUBIep* showed the most stability in geNorm. *CmRPL* also exhibited the most stability in NormFinder. Similarly, *CmUBC2* was recognized as the least stable gene by both of algorithms. Pairwise variation analysis showed that *CmUBIep*, *CmRPL*, *CmADP*, *CmRAN*, and *CmACT* (V_5/6_ = 0.149) were needed for more reliable normalization of gene expression in the organ of root (Fig. 2).

In the leaf subset (12 samples), *CmRAN* and *CmACT* showed the most stability in geNorm, and *CmADP* showed the most stability in NormFinder. Four genes, *CmRAN*, *CmACT*, *CmGAPC2*, and *CmADP* were needed for more reliable normalization in the leaf subset in geNorm pairwise variation analysis (V_4/5_ = 0.140) (Fig. 2).

In the abiotic stress subset (8 samples), *CmADP* was found to be the most stable gene by both geNorm (together with *CmRPL*) and NormFinder. Analysis of the pairwise variation showed *CmADP*, *CmRPL*, *CmRAN*, and *CmUBIep* were needed for more reliable normalization (V_4/5_ = 0.145) (Fig. 2).

In the biotic stress subset (6 samples), *CmTUA* and *CmACT* were ranked as the most stable genes in geNorm and *CmRPL* in NormFinder. The combination of *CmTUA*, *CmACT*, *CmRPL*, *CmUBIep*, and *CmRAN* was optimal for accurate normalization of gene expression (V_5/6_ = 0.139) (Fig. 2).

In the plant growth regulator treatment subset (7 samples), *CmRAN* and *CmACT* were found to be the most stable genes by geNorm. *CmADP* was determined to be the most stable gene by NormFinder. *CmRAN*, *CmACT*, *CmEF1α* and *CmGAPC2* (V_4/5_ = 0.149) were found to be the best genes for calculating the normalization factor in geNorm (Fig. 2).

### Expression profiles of catalase family genes

Using the Hidden Markov Model of catalase family, four genes (MELO3C017023, MELO3C017024, MELO3C026532, and MELO3C014643) were identified in the melon genome using HMMER with a threshold E-value<0.001. These genes were further confirmed by searching *Arabidopsis* proteins in TAIR database (http://www.arabidopsis.org/) with blastp. The genes MELO3C017023, MELO3C017024 and MELO3C026532 matched three members of *Arabidopsis* catalase gene family very closely. However, MELO3C014643 showed no significant match to any *Arabidopsis* proteins when a threshold E-value<1e-5 was used. For this reason, MELO3C014643 was dropped from further analysis. The other three genes are here called *CmCAT1*, *CmCAT2*, and *CmCAT3*, according to each gene's best match with its *Arabidopsis* counterpart. The primer pairs used for each gene in the melon catalase family were specifically designed to avoid amplification of multiple transcripts in the RT-qPCR analysis. Information regarding the identified melon catalase family genes and their primers is listed in Table S1. Meanwhile, integrity of the total RNA isolated from the root samples infected with Fusarium wilt was checked by electrophoresis in agarose gel. Results are shown in Fig. S3.

**Figure 3:**
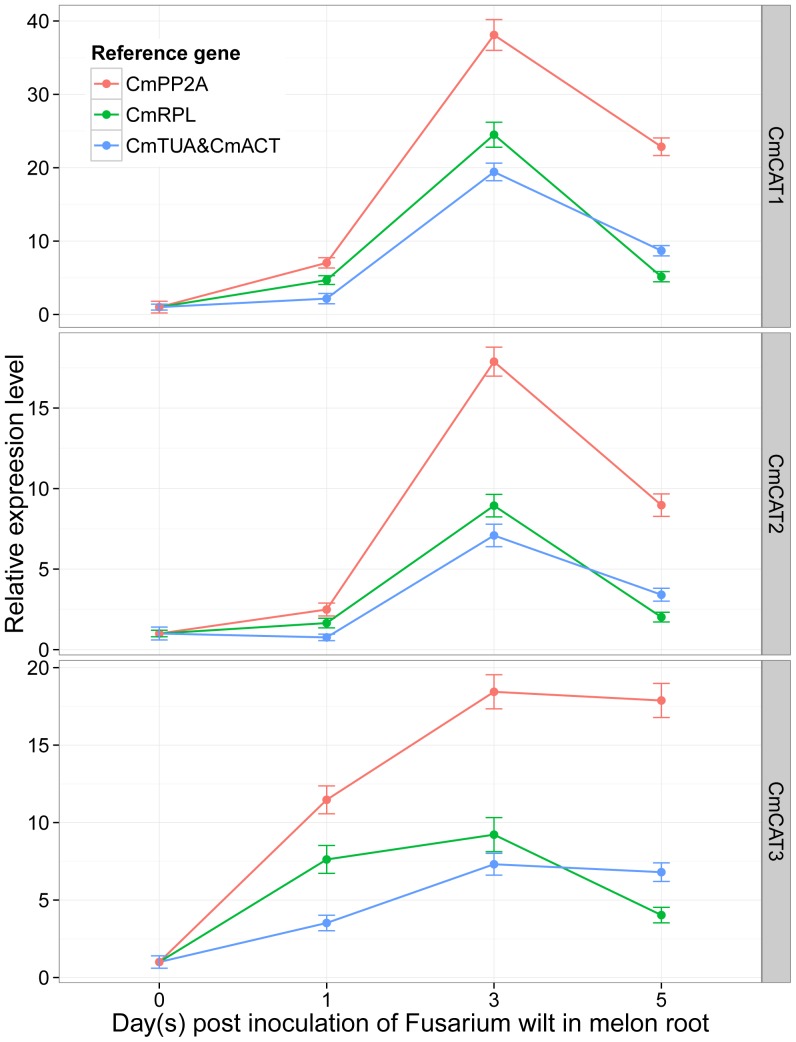
Expression profiles of melon catalase family genes in response to Fusarium wilt infection in roots. The relative expression levels of the catalase family genes were calculated as 2^−ΔΔCp^. The most stable genes identified by geNorm (*CmTUA* and *CmACT*) and NormFinder (*CmRPL*) and the least stable gene *CmPP2A* were used for normalization. For *CmTUA* and *CmACT*, the geometric mean was calculated and used for normalization. The relative expression levels are depicted as the mean±SD (standard deviation) calculated from three biological replicates.

geNorm identified *CmTUA* and *CmACT* as the genes that remained most stable when the plant was subjected to biotic stress, and NormFinder identified *CmRPL*. *CmPP2A* was found to be the least stable gene by both programs. To validate the reliability of the identified reference genes, the expression levels of melon catalase family genes were quantified using RT-qPCR and normalized using *CmRPL*, the combination *of CmTUA* and *CmACT*, and *CmPP2A* as reference genes. The results are presented in Fig. 3.

All the members of the melon catalase family genes were found to participate in the response to Fusarium wilt infection in roots. The average Cp values for *CmCAT1*, *CmCAT2*, and *CmCAT3* were 23.4, 25.6 and 19.9, respectively. Comparable expression levels were observed for the reference genes of *CmRPL* (22.5), *CmTUA* (23.3), *CmACT* (23.1), and *CmPP2A* (27.1). Similar patterns of expression were observed for melon catalase family genes. They were induced and upregulated to the highest level 3 days post inoculation and then downregulated. *CmCAT1* had a higher level of expression than *CmCAT2* or *CmCAT3*. The levels of expression of melon catalase family genes showed similar patterns of change, with only slight differences when the genes identified as the most stable by geNorm (*CmTUA* and *CmACT*) and NormFinder (*CmRPL*) were used for normalization. However, significantly higher expression levels were observed for these genes when the unstable reference gene *CmPP2A* was used for normalization. This led to overestimation of the levels of expression of catalase family genes. These results indicated that choice of reference gene can have a considerable impact on the normalization results and that using inappropriate reference genes may introduce bias to the analysis and cause misleading results.

## Discussion

Recently, the availability of the full sequence of the melon genome and multiple transcriptome data has inspired many diverse molecular biology studies performed in melons [2], [19], [31]–[34]. Because RT-qPCR is one of the most common approaches to functional genomics research, gene expression analysis involving RT-qPCR may become routine. The selection of suitable reference genes with stable expression is vital to effective normalization and the acquisition of accurate and meaningful biological data. It can also render data from different experiments comparable. However, in melons, systematic validation of reference genes received less attention and the traditional reference genes of *actin* or *cyclophilin* was frequently used without any appropriate validation, which probably hampered the accurate interpretation of the expression pattern of the target gene. Although *ubiquitin* has been validated for normalizing phosphorus responsive gene expressions in melon roots from 6 candidate housekeeping genes [1], the small number of the candidates has limited its ability to select the more suitable reference genes. Eleven reference genes were validated only in melon stems infected with Fusarium wilt in another study [21]. In the present study, 14 potential reference genes were selected and the stability of their expressions was assessed in leaves and roots subjected to biotic and abiotic stresses and to plant growth regulator treatments. These were used to screen appropriate reference genes for melon gene expression studies.

Many candidate reference genes, such as *actin*, *α-tubulin*, *translation elongation factor*, and *ADP-ribosylation factor*, belong to different families. Genes in the same family have conserved sequences. In the pre-genomic era, it was hard to design primers to specifically amplify one gene in a family because there was a dearth of sequence information regarding other members. This may cause simultaneous amplification of two or more genes in the same family when the primer pair is located in the conserved region. Different genes in the same family usually have different expression patterns. In wheat, the stability of expression of multi-transcript targeting references from the same family and single sequence amplification were compared in the gene families of *actin*, *tubulin*, *EF*, and *ADP*. The results proved that primer pairs targeting single gene for each of the analyzed families performed better than those targeting multiple genes [35]. In the present study, the melon genome information was used to design specific primers to each candidate, and genes from the same family were also evaluated for their expression stability. *CmACT* (*β-actin*) exhibited better expression stability than *CmACT7* as determined by both geNorm and NormFinder. *CmRPL* showed better performance than *CmRPL2*. These results show that using specific primer pair to amplify a single gene in a gene family for use as candidate reference is a very good strategy when the genome sequence is available.

Like other crops, melons showed wide ranges of expression abundance and variation with respect to the candidate reference genes [35], [36]. This indicated that no single gene was ideal for gene transcript normalization under the sets of conditions evaluated here. This proved that validation of commonly used reference genes is necessity. In general, the novel genes outperformed traditional reference genes with respect to expression stability under different treatments in the present study.

The most popular algorithms of geNorm and NormFinder were used to rank the candidate reference genes according to their stability of expression and to determine the number of reference genes necessary for accurate normalization of gene expression under the experimental conditions used here. However, the best reference genes as identified by geNorm and NormFinder in each sample subset showed several differences. Sometimes the two programs produce the same results and sometimes they do not. This has been observed in other crops [37], [38]. Because geNorm and NormFinder use distinct algorithms, different results can be expected. The two algorithms have their respective advantages. NormFinder takes the inter- and intra-group variations into account and is insensitive to co-regulating genes. geNorm can identify suitable combinations of reference genes required for normalization, which is more reliable than using single reference gene. Using both programs can render results more thorough. The use of both programs together is widespread in the selection of appropriate reference genes. Studies have also shown that the two algorithms are sufficient for the validation of reference genes [39]. In the present study, geNorm and NormFinder identified the same gene as the least stable in every experimental subset, *CmUBC2* in root and abiotic subsets and *CmPP2A* in the other subsets. Similar results have been observed in previous studies [38].

Different sample sets had their own best reference genes. For example, *CmADP* and *CmUBIep* were identified as the best reference genes across all samples. *CmUBIep* and *CmRPL* were identified as the most stable genes in roots. Similar results have been found for sweet potatoes and in the adventitious or lateral root development of poplars [40], [41]. *CmRAN* and *CmACT* were found to show the least variation in the leaf and plant growth regulator treatment subsets. *CmTUA* and *CmACT* were ranked as the most stable genes in the biotic stress subset and *CmADP* and *CmRPL* were ranked as the most stable genes in the abiotic stress subset. These results highlighted the importance of choosing reference genes appropriate to the experimental conditions in question. Because the variations in the expression of the selected genes, minimum reference gene set size of 4 to 6 genes is here needed for more reliable normalization. It is here recommended that organ-specific and experimental-condition-specific reference genes or the combination of multiple references be used for data normalization in gene expression analyses involving melons.

NormFinder identified *CmADP* as the best reference gene when all samples were taken into account and in leaf, abiotic stress, and plant growth regulator treatment subsets. *ADP* (*ADP-ribosylation factors 1*) is member of small GTP-binding proteins family and plays a critical role in intracellular trafficking, maintenance of endoplasmic reticulum morphology and plant growth [42], [43]. Similarly, *ADP* was found to be the most stable reference genes in the citrus rootstock “Swingle” citrumelo under drought stress conditions [44]. It was found to be the most stable gene in wheat and sweet potatoes in different tissues and different stages of development [40], [40]. Because *CmADP* has the most important characteristics of housekeeping genes, it may be a suitable candidate reference gene for use in the studies of melons.

Ribosomal proteins, the structural constituents of ribosomes, are ubiquitous in the plant kingdom. Several genes encoding ribosomal proteins have been successfully used as references in RT-qPCR analyses. In the present study, *CmRPL*, *CmRPL2*, and *CmRPL36aA* were also selected as the candidate reference genes. As in sweet potatoes, *CmRPL* was here ranked as the top reference gene by NormFinder in the root and biotic stress subsets [40]. However, ribosomal protein genes have shown tissue-dependent variations in mRNA expression and are not recommended for use as reference genes in animals [45].

The validation of reference genes has been carried out in several cucurbitaceous crops, including melons, cucumbers, and zucchinis. In cucumbers, *EF1α*, *Fbox*, *CAC* and *TIP41* have been demonstrated to be stable genes under different abiotic stresses, growth regulator treatments, and nitrogen nutrition situations [46], [47]. In another study on cucumbers, *EF1α*, *UBIep*, and *TUA* were also shown to be the most stable [48]. However, studies in zucchini showed that the combination of *UFP*, *EF1α*, *RPL36aA*, *PP2A*, and *CAC* genes was the best strategy for reliable normalization [3]. *RPL2*, *ACT*, and *cyclophilin* were found to be the best three reference genes in melon stems infected with Fusarium wilt [21]. In the present study, the combination of *CmADP* and *CmUBIep*, *CmRPL*, *CmRAN*, *CmACT*, and *CmEF1α* was found to be a suitable multiple reference set for normalization of gene expression in all samples evaluated using geNorm. The optimal reference genes identified in melons were different from those identified in cucumbers and zucchinis, suggesting that identification of the most stable reference genes in each species is essential. The best reference genes identified in melon roots and leaves in the present study were also different from those identified in melon stems [21]. This further highlighted the organ-specific characteristic of these reference genes. Accordingly, it is necessary to systematically validate the stability of the expression of candidate reference genes prior to their use in RT-qPCR normalization.

Currently, *actin* was the most widely used reference gene in melons [12]–[18]. However, in the present study this gene was not found to be the best internal control under most experimental conditions by geNorm or under any set of experimental conditions by NormFinder. *Actin* was also found to vary considerably in many other crops [37], [49]. Recently, it has been suggested that *actin* should not be used as a reference in RT-qPCR analysis because of high number of pseudogenes on the genome. These pseudogenes could be amplified if the primers are not sufficiently specific [50]. It is here suggested that *actin* not be used as reference gene in melons or at least used alongside other reference genes in RT-qPCR analysis. The traditional housekeeping genes *cyclophilin* and *ubiquitin* have also been used in melons [1], [19], [20]. Because novel reference genes usually outperform traditional ones, a great deal of attention was paid to screening out the novel reference genes, so these two genes were not taken into consideration in the present study. *Actin*, *cyclophilin*, and *ubiquitin* were also found to be less stable than other genes in cucumbers [46].

Catalase family genes, the major scavengers of H_2_O_2_ under adverse stress conditions, were identified in the melon genome and used as target genes to test the impact of reference genes with different levels of stability on the results of normalization. All members of the catalase family genes were evaluated in response to Fusarium wilt infection in melon roots. The facts that they had similar expression patterns and that *CmCAT1* had higher levels of expression than *CmCAT2* or *CmCAT3* suggested that *CmCAT1* was an important player in the removal of H_2_O_2_ generated under Fusarium wilt stress, and that *CmCAT2* and Cm*CAT3* were major H_2_O_2_ scavengers contributing to ROS homeostasis [51]. When the least stable gene *CmPP2A* was used as reference gene for normalization, the levels of catalase family gene expression were significantly overestimated. Those results highlighted the importance of selecting suitable reference genes for normalization in RT-qPCR analysis.

This is the first systematic validation of suitable reference genes in melon roots and leaves and identification of catalase family genes in melon genome. These results can be used as guidelines for the selection of reference genes for use in analyses of gene expression. This may benefit future studies that require more accurate gene expression quantification. They also provide valuable information for the study of the catalase gene family in melons. However, it should be pointed out that the large and constantly growing number of melon expression profiling data may offer an excellent source for identification of novel and more stable reference genes. These deserve attention and should be mined in the future.

## Supporting Information

Figure S1 **PCR amplification patterns of candidate reference genes in melon on cDNA and genomic DNA templates.** “c” represents the cDNA template. “g” represents the genomic DNA template. “M” represents markers of 50 bp ladders (Tiangen). Genomic DNA was isolated from the leaves using a Plant Genomic DNA Kit (Tiangen). PCR amplifications were conducted using 2×PCR Reagent (Tiangen) according to the manual. The amplification products were resolved on 2% agarose gel for 30 min at 120 V.
(PDF)

Figure S2 **Melting curve analyses of melon candidate reference genes.**
(PDF)

Figure S3 **Integrity of the total RNA isolated from the root samples infected with Fusarium wilt confirmed by electrophoresis in 2% agarose gel.** DPI represents day(s) post inoculation. The numbers 1, 2, and 3 represent the three biological replicates. “M” represents the DL 2000 DNA marker (TaKaRa).
(PDF)

Table S1 Descriptions of melon catalase family genes.
(PDF)
