# Supplementary material for: Screening Suitable Reference Genes for Normalization in Reverse Transcription Quantitative Real-Time PCR Analysis in Melon
Source: PLoS One. 2014 Jan 27;9(1):e87197. doi: 10.1371/journal.pone.0087197 (PMC3903635; doi:10.1371/journal.pone.0087197)
Supplement: Figure S1 — PCR amplification patterns of candidate reference genes in melon on cDNA and genomic DNA templates. “c” represents the cDNA template. “g” represents the genomic DNA template. “M” represents markers of 50 bp ladders (Tiangen). Genomic DNA was isolated from the leaves using a Plant Genomic DNA Kit (Tiangen). PCR amplifications were conducted using 2×PCR Reagent (Tiangen) according to the manual. The amplification products were resolved on 2% agarose gel for 30 min at 120 V. (PDF) [file pone.0087197.s001.pdf]

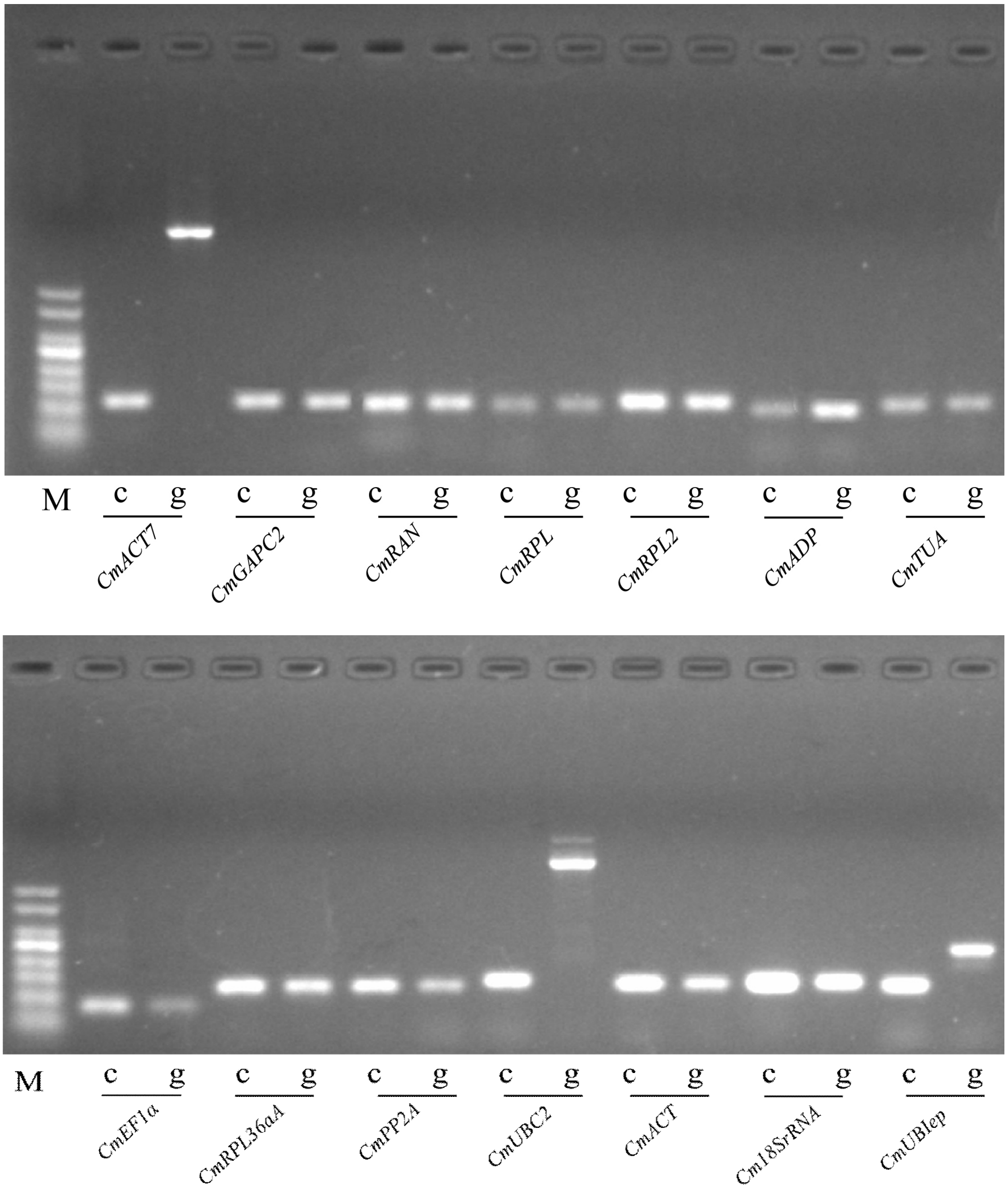

Figure S1. PCR amplification patterns of candidate reference genes in melon on cDNA and genomic DNA templates. "c" represents the cDNA template. "g" represents the genomic DNA template. "M" represents markers of 50 bp ladders (Tiangen). Genomic DNA was isolated from the leaves using a Plant Genomic DNA Kit (Tiangen). PCR amplifications were conducted using 2×PCR Reagent (Tiangen) according to the manual. The amplification products were resolved on 2% agarose gel for 30 min at 120 V.
