# Supplementary figures and images for: Screening Suitable Reference Genes for Normalization in Reverse Transcription Quantitative Real-Time PCR Analysis in Melon
Source: PLoS One. 2014 Jan 27;9(1):e87197. doi: 10.1371/journal.pone.0087197 (PMC3903635; doi:10.1371/journal.pone.0087197)

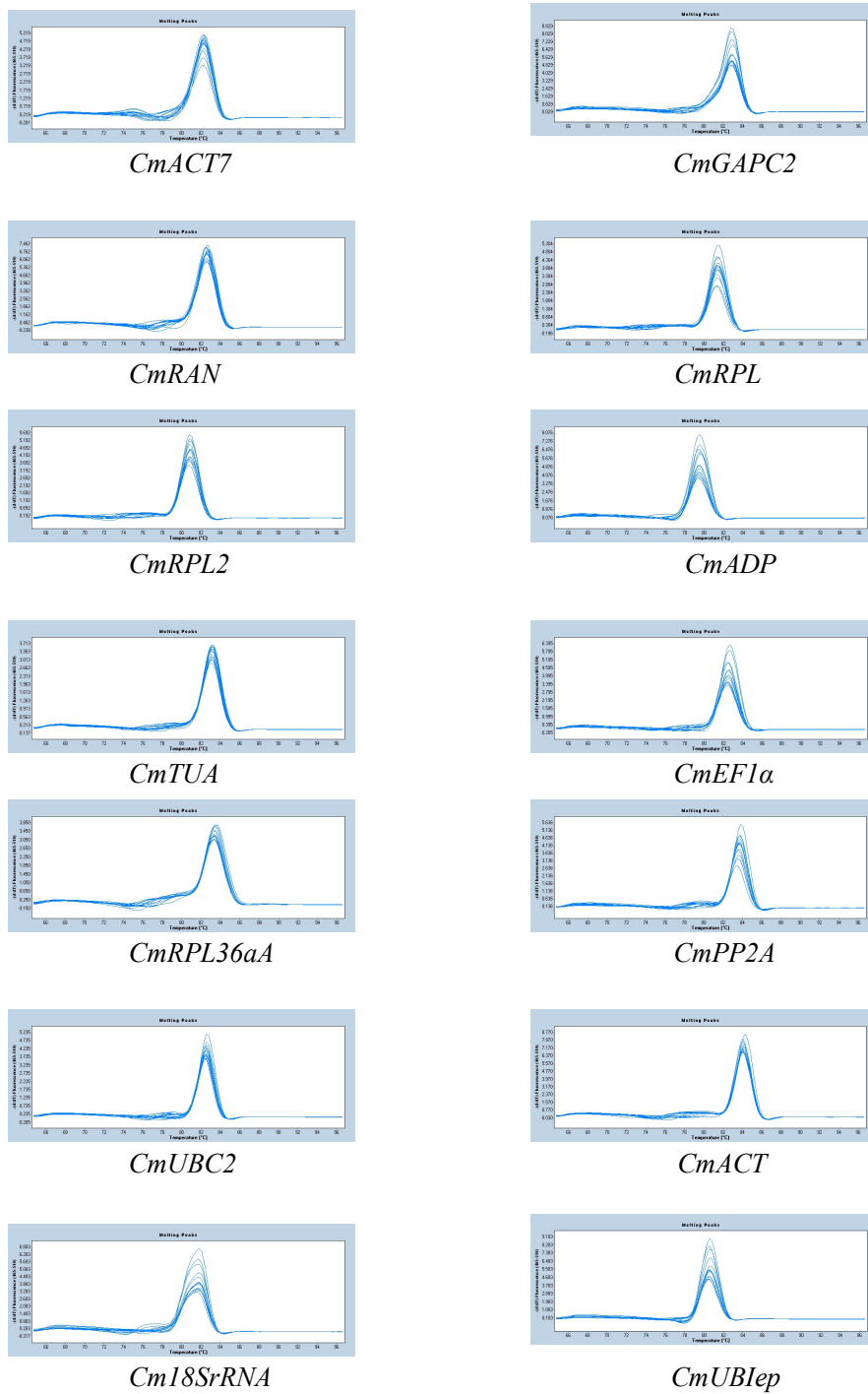

**Figure S2. Melting curve analyses of melon candidate reference genes.**

Supplement: Figure S2 — Melting curve analyses of melon candidate reference genes. (PDF) [file pone.0087197.s002.pdf]
