# Supplementary material for: Screening Suitable Reference Genes for Normalization in Reverse Transcription Quantitative Real-Time PCR Analysis in Melon
Source: PLoS One. 2014 Jan 27;9(1):e87197. doi: 10.1371/journal.pone.0087197 (PMC3903635; doi:10.1371/journal.pone.0087197)
Supplement: Figure S3 — Integrity of the total RNA isolated from the root samples infected with Fusarium wilt confirmed by electrophoresis in 2% agarose gel. DPI represents day(s) post inoculation. The numbers 1, 2, and 3 represent the three biological replicates. “M” represents the DL 2000 DNA marker (TaKaRa). (PDF) [file pone.0087197.s003.pdf]

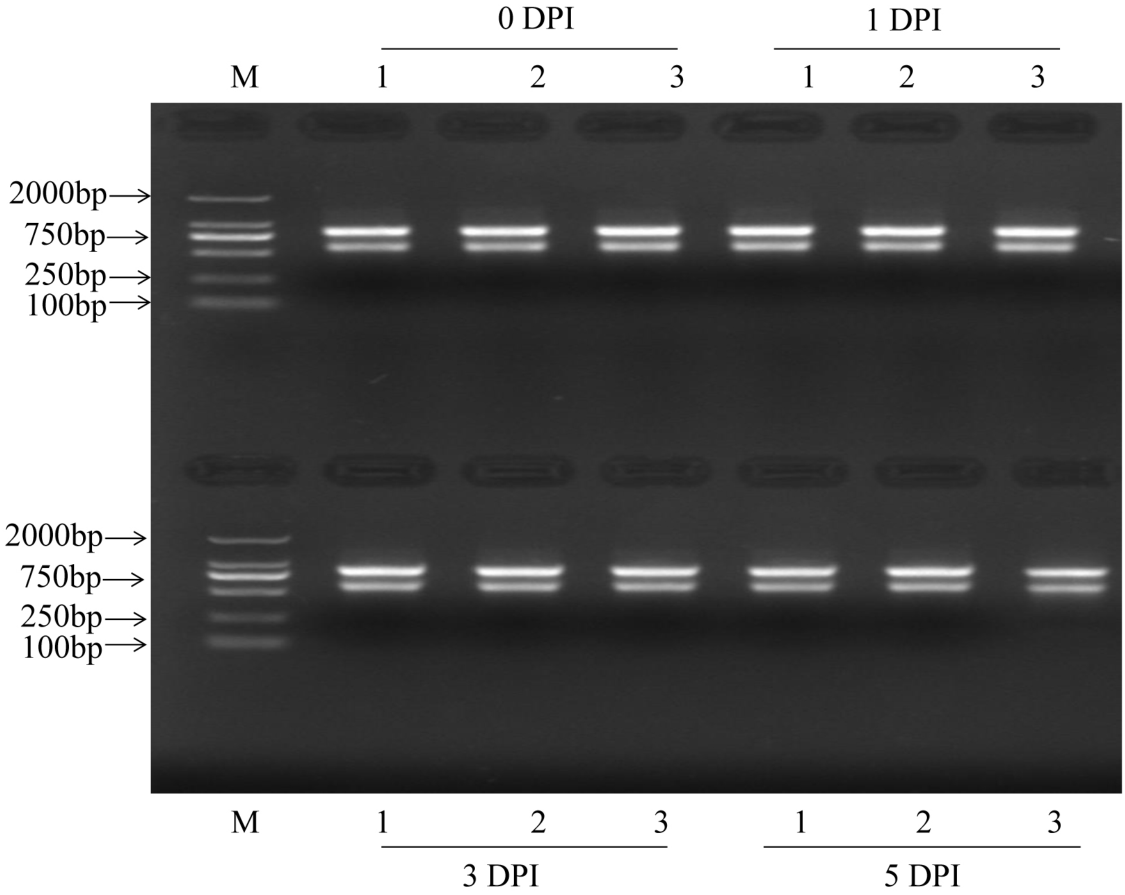

Figure S3. Integrity of the total RNA isolated from the root samples infected with Fusarium wilt confirmed by electrophoresis in 2% agarose gel. DPI represents day(s) post inoculation. The numbers 1, 2, and 3 represent the three biological replicates. "M" represents the DL 2000 DNA marker (TaKaRa).
