# Supplementary material for: Screening Suitable Reference Genes for Normalization in Reverse Transcription Quantitative Real-Time PCR Analysis in Melon
Source: PLoS One. 2014 Jan 27;9(1):e87197. doi: 10.1371/journal.pone.0087197 (PMC3903635; doi:10.1371/journal.pone.0087197)
Supplement: Table S1 — Descriptions of melon catalase family genes. (PDF) [file pone.0087197.s004.pdf]

Table S1. Descriptions of melon catalase family genes

Gene name	Gene ID[1]	Arabidopsis homolog[2]	E-value	Identity[3]	Primer sequence[4]	Product size (bp)
CmCAT1	MELO3C017024	AT1G20630	0	87%	F: TCTGGTGCTCCTGTGTGGAAC<br>R: GCTCTGGAATCCGCTCTCTATC	124
CmCAT2	MELO3C017023	AT4G35090	0	92%	F:TCCTTACAGGCACCGACCTTC<br>R: ACGGTCAACGAGGAGTTGTTG	96
CmCAT3	MELO3C026532	AT1G20620	0	75%	F:CTCTTCTCTCCACCGCCATG<br>R:TTCTCCGACGGACATTACGG	125

"1": Melon gene ID in Melonomic database (https://melonomics.net/genome/);

"2": Arabidopsis gene ID in TAIR database (http://www.arabidopsis.org/);

"3": The identity data are the results of blastp;

"4": F, forward primer; R, reverse primer.
